# Entrepreneurial effect of rural return migrants: Evidence from China

**DOI:** 10.3389/fpsyg.2022.1078199

**Published:** 2022-12-22

**Authors:** Anze Bao, Gefeng Pang, Guoping Zeng

**Affiliations:** ^1^School of Economics and Business Administration, Chongqing University, Chongqing, China; ^2^School of Public Policy and Administration, Chongqing University, Chongqing, China

**Keywords:** rural return migrants, entrepreneurship, land circulation, physical capital, human capital

## Abstract

Entrepreneurship is an important means of economic development. Rural migrant workers returning home to start their own businesses can promote employment, alleviate poverty, and achieve rural development structural transformation of rural development. The entrepreneurial effect of rural return migrants is important for rural economic development. Using the data of the China Labor Force Dynamics Survey (CLDS thereafter) 2018 and China Household Finance Survey (CHFS thereafter) 2019, we analyze the entrepreneurial effects of return migrants upon their return to their hometowns. We construct a career choice model and build a mathematical model based on it to formulate the hypothesis. Then, we use the Probit regression model to test the hypothesis empirically. Results find that the rural return migrants can promote entrepreneurship among residents. Land circulation, human capital, and physical capital are stimulating factors in promoting the rural entrepreneurial activities of return migrants. We recommend that the government actively guide the rural return migrants to start businesses and provide security for entrepreneurial activities by upgrading various entrepreneurial elements.

## 1. Introduction

Entrepreneurial activities are conducive to promoting innovation, enhancing market competition, and creating jobs. As China’s economy shifts from high growth to high-quality development, creating an entrepreneurial and innovative ecology has become an urgent requirement for economic development (He et al., 2019; Li D. et al., 2022). With the promotion of national entrepreneurship policies and the encouragement of local entrepreneurship education, entrepreneurial activities are becoming increasingly active and the number of entrepreneurial enterprises is growing rapidly. In entrepreneurial activities, the participation of farmers has gradually increased, becoming an important part of the current entrepreneurial community (Miao et al., 2021). Entrepreneurship of farmers helps to drive the employment of surplus rural labor and alleviate the current employment difficulties. It also increases farmers’ income and promotes rural revitalization and prosperity (Naminse et al., 2018).

Since the opening-up reform of China in 1978, the government has actively promoted industrialization and urbanization, encouraging surplus labor from rural areas to move to urban areas for employment to coordinate urban–rural integration development (Liang and Morooka, 2004; Liu et al., 2015). Meanwhile, there are always many migrants returning to the countryside due to the “pull” or “push,” making career choices between starting a business, working, or farming (Ma, 2002; Jia and Liu, 2014). Driven by the entrepreneurship policy, the government actively encourages migrant workers to return to their hometowns and engage in entrepreneurship, to achieve sustainable development in rural areas. Compared to farmers staying in the local area, returning entrepreneurs have more advantages in economic, human, and social capital and play a leading role in promoting transformation, upgrading the rural industries, and promoting employment in rural areas (Naminse et al., 2018). Therefore, the rural return migrants can realize the entrepreneurial effect is important for the government to formulate scientific entrepreneurship and rural development policies.

Rural return migrants also bring with them the movement of various production factors such as knowledge, skills, and capital. These factor flows to optimize the conditions for residents to start their own businesses and influence their career choices. In the process of going out to work, people are able to learn knowledge and exercise skills. Rural return migrants bring knowledge and skills back to their hometowns, driving the flow of human capital. In addition, the main purpose of working outside the home is to obtain higher returns and accumulate wealth, which provides the basis for rural return migrants to start their own businesses (Yang and Wen, 2020). Meanwhile, the choice of rural return migrants to work outside, especially on a long-term basis, often means people would give up their land holdings, which will further lead to the transfer of land (Deininger and Jin, 2009). Rural return migrants who return to the countryside are more likely to start their own businesses because of the relatively small amount of land available (Barth and Zalkat, 2021).

Existing literature on entrepreneurship focuses on the factors and consequences. The factors that influence entrepreneurship focus on individual, social, and policy aspects. Individual factors include education, risk appetite, occupation experience, and family background (Halvarsson et al., 2018; Duleep et al., 2022; Giacomin et al., 2022). Social factors refer to migration and mobility (Lee and Eesley, 2018; Duleep et al., 2022), the internet economy and digital finance (Cumming and Johan, 2010; Liu et al., 2022), and the business environment (Wu and Lin, 2021). Entrepreneurial support policies such as tax relief (Darnihamedani et al., 2018; Audretsch et al., 2022), government decentralization (Rangus and Slavec, 2017; Zeng and Wen, 2021), and approval process simplification (Li et al., 2021; Shi and Frenkiel, 2021). Entrepreneurial support policies are also influencing entrepreneurial choices. However, none of the above studies has examined the entrepreneurial effects of rural return migrants.

Rare literature refers to the entrepreneurial effects of the rural return migrants. Although some scholars have started to focus on this area, they have focused more on migrants who leave the countryside to work and tried to explain how the rural return migrants contribute to resident entrepreneurship (Ma, 2002; Démurger and Xu, 2011; Jia and Liu, 2014; Jia et al., 2017; Oostendorp, 2017; Zhou et al., 2017; Wang et al., 2018; Li Y. et al., 2022; Wu et al., 2022). But these do not answer the question of whether and how the rural return migrants impact rural entrepreneurship.

From the above analysis, existing literature mainly focuses on the economic effects of rural return migrants, but less discusses the entrepreneurial effects. We attempt to answer the question “whether rural return migrants affect entrepreneurship” from both theoretical model and empirical analysis. We first construct a model of occupational choice to analyze the different returns to residents engaging in entrepreneurship, working, and farming, as well as the occupational choices made under changing resource endowments and constraints. Based on the mathematical model, we propose a hypothesis that the rural return migrants affect entrepreneurship through land circulation human capital and physical capital. Then, we use China Labor Force Dynamics Survey (CLDS) (2018) and China Household Finance Survey (CHFS) (2019) to empirically test the entrepreneurial effects of rural return migrants. We find rural return migrants have a positive effect on entrepreneurship, and this positive effect holds when controlling for the remaining variables. The findings still hold when considering endogeneity. We further find that the entrepreneurial effect of the rural return migrants is mainly formed through land circulation, human capital, and physical capital.

This study examines the return of rural labor to their hometowns in the context of current economic development, and the results are important for world economic development. Whether or not rural migrants choose to return to their hometowns is ostensibly guided by policy and economic development but is actually the result of human psychological and behavioral choices. The contributions of this paper mainly include: First, existing studies have mainly focused on the migration of migrant workers from rural to urban areas (Zhang and Wang, 2010; Lan, 2014; Chen and Liu, 2016; Roberts, 2018; Ge et al., 2020; Qiao et al., 2022), with few studies on the economic consequences of rural return migrants and studies on their entrepreneurial effect. We construct a theoretical framework for the analysis of the impact of rural return migrants on entrepreneurship to find the practical basis and enrich the existing rural labor mobility, economic growth, and psychological theories from a new perspective. Second, we focus on the indirect mechanism of rural return migrants affecting residents’ entrepreneurship from human capital, physical capital, and land circulation. This series of factors reflects the impact of the economic development environment on psychological and behavioral choices, which complements the research in the field of psychology and economics. It further expands the mechanism by which the rural return migrants influence entrepreneurship, and thus makes the dynamic mechanism research clearer. In addition, we focus on the trajectory of rural return migrants and their impact on entrepreneurial behavior. Our findings contribute to allocating rural labor resources scientifically and rationally and improving the efficiency of economic activities and promoting the quality transformation of the current economy. This is particularly relevant to the declining birth rates and the increasing aging of the population worldwide.

The remainder of the paper proceeds as follows. Section 2 “Theoretical models and hypotheses” covers the theoretical models and hypotheses. Section 3 “Methodology” presents the methodology. Section 4 “Results” outlines the results and discussion. Section 5 “Mechanisms” adopts a mechanisms analysis. Finally, section 6 “Conclusion and implications” summarizes the main conclusions and implications.

## 2. Theoretical models and hypotheses

### 2.1 Assumptions

To analyze the occupational choice behavior of the population through a mathematical model, we make the following theoretical assumptions:

i. People are rational and make career choices based on maximizing two-phase utility.

ii. Utility satisfies the general utility function *u*(⋅), *u*′(⋅)> 0, and u(⋅)″<0, when the dependent variable is consumer funds.

iii. Individuals have different endowments and preferences. Specifically, (a) physical capital (ω_*i*_), where individuals have different initial capital due to objective constraints, and income and spending power. (b) discount rate (β_*i*_), where the discount rate varies due to different risk appetites and expected returns. (c) loan limits $\left({\bar{b}}_{i}\right)$, where credit conditions, social connections, and other aspects possessed by individuals result in differences in the ability to access funds and different maximum loan amounts.

iv. Workers will receive wages, the farmer will receive income from agricultural product sales, and entrepreneurs will receive the return from entrepreneurship if they succeed or obtain residual if they fail. *w*_*i*_ denotes wage, determined by human capital. *f*_*i*_ measures output per unit of land. *p* is the probability of entrepreneurial success. The entrepreneurs receive the return of *y*_*s*_ for a successful venture and otherwise *y*_*f*_.

v. The model only discusses the current and future periods. As income from the second and infinite subsequent periods can be discounted to the second period, this is reflected mathematically as a change in the discount rate in the second period. Also, the model does not rely on the specific setting of the discount rate.

vi. The impact of job promotion and investment volatility is not considered. As only two periods of returns are discussed, the case of appreciation and investment volatility can be excluded and as they are not the focus of this paper, ignoring the case of appreciation and investment volatility has the effect of simplifying the model.

vi. The model does not include the impact of job promotions and investment fluctuations. The model only discusses two-period returns, and thus excludes job promotions and investment fluctuations.

The variables involved in the model and their definition are shown in Supplementary Appendix 1.

### 2.2 Occupational option models

We consider a simple two-period career choice model: individuals can autonomously allocate resources in the first period, while the initial resource allocation will determine the total utility in the future. Initial resource allocation aims to maximize the total utility. The total utility includes two components: (i) current consumption utility, and (ii) discounted future potential utility. Specifically, residents can determine their loan (*b*_*i*_) and investment amount (*m*_*i*_) in the first period, which will further determine their consumption in both periods (*x*_*i*_, *y*_*i*_). Total utility is a function of consumption. Therefore, we can determine the optimal loan ($b_{i}^{*}$) and investment ($m_{i}^{*}$) to maximize total utility (*U*_*i*_ = *V*_*i*_). By comparing the total utility from different career choices, it will select the career with the highest total utility. Residents make decisions based on the utility of entrepreneurship.

#### 2.2.1 Entrepreneurial returns

First, we discuss the returns to resident entrepreneurship. Entrepreneurs aim to maximize two-period utility, so we use Eq. (1) to measure the total utility of entrepreneurship and satisfy the constraints.


$$\text{max}\,U_{e\,i}\,\left(x_{i},y_{s,i},y_{f,i}\right)$$

$$=u\,\left(x_{e,i}\right)+\beta \,\left[p\,u\,\left(y_{s,i}\right)+\left(1-p\right)\,u\,\left(y_{f,i}\right)\right]=u_{e,1}\,\left(\omega_{i},b_{i},m_{i}\right)$$

(1) $$+\beta \,\left[p\,u_{e,2}\,\left(R_{i},r,b_{i},m_{i}\right)+\left(1-p\right)\,u_{e,3}\,\left(\emptyset ,r,b_{i}\right)\right]$$

$$s.t.x_{e,i}=\omega_{i}+b_{e,i}-m_{i}s.t.x_{e,i}=\omega_{i}+b_{e,i}-m_{i}$$

$$y_{s,i}=R_{i}\,m_{i}-r\,b_{e,i}y_{s,i}=R_{i}\,m_{i}-r\,b_{e,i}$$

$$y_{f,i}=\emptyset -b_{e,i}y_{f,i}=\emptyset -b_{e,i}$$

$$b_{e,i}\leq {\bar{b}}_{i}\,0\leq x_{e,i},\,y_{s,i},y_{f,i}b_{e,i}\leq {\bar{b}}_{i}0\leq x_{e,i},\,y_{s,i},y_{f,i}$$

In Eq. (1), we believe the total individual utility includes two components: (i) current utility, and (ii) expected entrepreneurship utility. β is the discount rate,β ∈ (0.1). *p* is the probability of success of the venture, *p* ∈ (0.1). The success of entrepreneurship is influenced by the external environment. When the resident succeeds in starting a business, the funds used for consumption are *y*_*s,i*_, otherwise *y*_*f,i*_. Note that since u(⋅)″<0, the expected utility of consumption here is not equal to the utility of expected consumption.

For the constraints, the first three equations are the consumption. Consumption is the original capital accumulation ω_*i*_, plus the loan *b*_*e,i*_, and minus the entrepreneurial capital *m*_*i*_, as entrepreneurs have invested and taken out a loan in the first period. In the second period, the return *y*_*s,i*_ is the entrepreneurial return minus the repayment amount in case of a successful start-up, or no start-up return under a failed start-up. *R*_*i*_ is the investment return and measures entrepreneurial talents. The more talented the entrepreneur, the higher the return on investment. Generally, the benefits of entrepreneurship outweigh the costs (*R*_*i*_ > 1). We also assume only residuals ∅ remain after investment failure. The loan has to be repaid regardless of the outcome of the investment. *r* is the borrowing rate, and therefore, the consumption amount less *rb*_*i*_ in the second period. Therefore, if the investment is successful, consumption in the second period is the investment return minus the loan repayment. Conversely, the investment residual minus the loan amounts.

The fourth constraint indicates that an individual cannot borrow more than the maximum loan amount he or she can obtain through various sources ${\bar{b}}_{i}$. The fifth constraint means that people invest rationally and will ensure their most basic survival needs whether they succeed or not. *x*_*e*,*i*_, *y*_*s*,*i*_, and *y*_*f,i*_ are all positive.

Therefore, we can determine the indirect utility function *V*_*e,i*_ for the entrepreneur’s optimal loan ($b_{e,i}^{*}$) and investment ($m_{i}^{*}$) based on individual conditions. Rational individuals will self-select the optimal amount. The choice of loan and investment is not the focus of this study, thus, the specific values and expressions are not derived.

Optimal loan amount. We make Eq. (2) equal to zero to obtain the individual optimal amount of maximum utility $b_{e,i}^{*}$. Since there is a range restriction, if $b_{e,i}^{*}$ > ${\bar{b}}_{i}$, then let $b_{e,i}^{*}$ = ${\bar{b}}_{i}$. $U_{e\,i}^{{}^{\prime }}\,\left(b_{e,i}^{*}\right)$ > 0 when the above case is satisfied, but *U*_*ei*_ is also the utility at the optimal loan amount:


$$U_{e\,i}^{\prime }\,\left(b_{e,i}^{*}\right)=\frac{\partial U_{e\,i}}{\partial b_{e,i}}=\frac{\partial u\,\left(x_{e,i}\right)}{\partial b_{e,i}}+p\,\beta \,\frac{\partial u\,\left(y_{s,i}\right)}{\partial b_{e,i}}$$

(2) $$+\left(1-p\right)\,\beta \,\frac{\partial u\,\left(y_{f,i}\right)}{\partial b_{e,i}}\geq 0$$

Optimal entrepreneurial amount. Similar to the above, let $U^{\prime }\,\left(m_{i}^{*}\right)$ = 0, to obtain the optimal investment amount $m_{i}^{*}$. Although *m*_*i*_ ∈ [0,ω_*i*_ + *b*_*i*_], since u(⋅)″ < 0, $m_{i}^{*}$ must fall within the interval. Thus, $U_{e\,i}^{\prime }\,\left(m_{i}^{*}\right)$=0 must hold, satisfying Eq. (3):


(3) $$U_{e\,i}^{\prime }\,\left(m_{i}^{*}\right)=\frac{\partial U_{e\,i}}{\partial m_{i}}=\frac{\partial u\,\left(x_{e,i}\right)}{\partial m_{i}}+p\,\beta \,\frac{\partial u\,\left(y_{s,i}\right)}{\partial m_{i}}=0$$

The indirect utility function (*V*_*ei*_) of the individual corresponding to the optimal personal loan$\left(b_{e,i}^{*}\right)$ and investment $\left(m_{i}^{*}\right)$ derived from Eq. (2) and Eq. (3), is shown in Eq. (4):


$$V_{e\,i}\,\left(\omega_{i},R_{i},r,\emptyset \right)$$

$$=u_{e,1}\left(\omega_{i},b_{e,i}^{*},m_{i}^{*}\right)+\beta [pu_{e,2}\left(R_{i},r,b_{e,i}^{*},m_{i}^{*}\right)$$

(4) $$+\left(1-p\right)u_{e,3}\left(\emptyset ,r,b_{e,i}^{*}\right)]$$

After determining loans and investments, the maximum utility *U*_*ei*_ can be represented by the *V*_*ei*_ in Eq. (4), determined only by the individual (ω_*i*_,*R*_*i*_) and environmental characteristics(*r*,∅).

#### 2.2.2 Work returns

Similar to entrepreneurial returns, work returns are also affected by the discounting of the first and the second period utility, and the consumer still aims to maximize both periods’ utility as shown in Eq. (5). The difference is that the effect of the second period utility as a result of wage. We assume wage *w*_*i*_to be stable, thus, the utility from the wage is also stable:


$$\text{max}\,U_{w\,i}\,\left(x_{i},y_{i}\right)=u\,\left(x_{w,i}\right)+\beta \,u\,\left(y_{w,i}\right)$$

(5) $$=u_{w,1}\,\left(\omega_{i},b_{w,i}\right)+\beta \,u_{w,2}\,\left(w_{i},b_{w,i},r\right)$$

$$s.t.x_{w,i}=\omega_{i}+b_{w,i}s.t.x_{w,i}=\omega_{i}+b_{w,i}$$

$$y_{w,i}=w_{i}-r\,b_{w,i}y_{w,i}=w_{i}-r\,b_{w,i}$$

$$b_{w,i}\leq {\bar{b}}_{i}\,0\leq x_{w,i},\,y_{w,i},y_{w,i}b_{w,i}\leq {\bar{b}}_{i}0\leq x_{w,i},\,y_{w,i},y_{w,i}$$

Since there is no investment in the utility of the first period, *x*_*w*,*i*_ = ω_*i*_ + *b*_*w*,*i*_. However, it could allocate capital across periods using loans. When *b*_*w*,*i*_ > 0, the individual uses a loan for consumption. Conversely, when *b*_*w*,*i*_ < 0, the individual deposits part of the current capital for consumption in the next period and receive interest *r*. Thus, two-period income includes the impact from borrowings and loan repayments. Wage remains unchanged, so consumption in the second period can be expressed as *w*_*i*_−*rb*_*w*,*i*_. The remaining restrictions are the same as in the case of entrepreneurial returns.

First, we determine the optimal loan. The utility of the worker is also affected by the loan (*b*_*w*,*i*_). Individuals can allocate funds across time with different loan amounts. We can obtain the optimal loan $b_{w,i}^{*}$ when the first-order partial derivative is 0, and $b_{w,i}^{*}$ satisfies $U^{\prime }\,\left(b_{w,i}^{*}\right)\geq 0$ [refer to Eq. (6)]. Specifically, when $b_{w,i}^{*}\in \left[0,{\bar{b}}_{i}\right]$, $U^{\prime }\,\left(b_{w,i}^{*}\right)=0$. Otherwise, $U^{\prime }\,\left(b_{w,i}^{*}\right)>0$ and $b_{w,i}^{*}$ are still the optimal loan:


(6) $$U_{w\,i}^{\prime }\,\left(x_{i},y_{i}\right)=\frac{\partial U_{w\,i}}{\partial b_{w,i}}=\frac{\partial u\,\left(x_{w,i}\right)}{\partial b_{w,i}}+\beta \,\frac{\partial u\,\left(y_{w,i}\right)}{\partial b_{w,i}}\geq 0$$

Then, we believe that since the worker does not invest, his indirect utility function follows Eq. (7). The maximum utility of the worker is limited by the ω_*i*_, *w*_*i*_, and *r*:


(7) $$V_{w\,i}\,\left(\omega_{i},w_{i},r\right)=u_{w,1}\,\left(\omega_{i},b_{w,i}^{*}\right)+\beta \,u_{w,2}\,\left(w_{i},b_{w,i}^{*},r\right)$$

#### 2.2.3 Farming returns

In addition to entrepreneurship and work, rural return migrants can also choose to engage in agricultural activities. Similar to entrepreneurship and work returns, we can obtain farming returns of rural residents and the corresponding two-period maximum utility using Eq. (8). Variables are the same as in the previous section. The difference is that farming returns are determined by the area of arable land owned by the rural resident and the agricultural production level. *A*_*i*_ is the arable land the rural resident owns, and *f*_*i*_ is the agricultural production level of the individual, which measures output per unit of land:


$$\text{max}\,U_{a\,i}\,\left(x_{i},y_{i}\right)=u\,\left(x_{a,i}\right)+\beta \,u\,\left(y_{a,i}\right)$$

(8) $$=u_{a,1}\,\left(\omega_{i},b_{w,i}\right)+\beta \,u_{a,2}\,\left(f_{i},A_{i},b_{a,i},r\right)$$

$$s.t.x_{a,i}=\omega_{i}+b_{a,i}s.t.x_{a,i}=\omega_{i}+b_{a,i}$$

$$y_{a,i}=f_{i}\,A_{i}-r\,b_{a,i}y_{a,i}=f_{i}\,A_{i}-r\,b_{a,i}$$

$$b_{a,i}\leq {\bar{b}}_{i}b_{a,i}\leq {\bar{b}}_{i}$$

$$A_{i}\leq \,{\bar{A}}_{i}\,0\leq x_{a,i},\,y_{a,i},A_{i},b_{a,i}A_{i}\leq \,{\bar{A}}_{i}0\leq x_{a,i},\,y_{a,i},A_{i},b_{a,i}$$

Since farmers do not invest, the consumption of farmers in the first period is the same as that of workers. Farmers can only allocate funds through savings and loans, denoted by ω_*i*_ + *b*_*a*,*i*_. Consumption in the second period is farm income minus loan (*f*_*i*_*A*_*i*_−*rb*_*a*,*i*_), where farm income is the product of the land owned amount (*A*_*i*_) and output value per unit of land(*f*_*i*_).

First, we determine the optimal loan. Farmers allocate funds across time based on loans (*b*_*a*,*i*_). We can obtain the optimal loan $b_{a,i}^{*}$ when the first-order partial derivative is 0, and $b_{a,i}^{*}$ satisfies $U^{\prime }\,\left(b_{a,i}^{*}\right)\geq 0$ [refer to Eq. (9)]. Specifically, when $b_{a,i}^{*}\in \left[0,{\bar{b}}_{i}\right]$, $U^{\prime }\,\left(b_{a,i}^{*}\right)=0$. Otherwise, $U^{\prime }\,\left(b_{a,i}^{*}\right)>0$ and $b_{a,i}^{*}$ are still the optimal loan:


(9) $$U_{a\,i}^{\prime }\,\left(b_{a,i}\right)=\frac{\partial U_{a\,i}}{\partial b_{a,i}}=\frac{\partial u\,\left(x_{a,i}\right)}{\partial b_{a,i}}+\beta \,\frac{\partial u\,\left(y_{a,i}\right)}{\partial b_{a,i}}\geq 0$$

Then, we present the indirect utility of the farmer in Eq. (10). The maximum utility of the farmer is determined by ω_*i*__‵_ *r*, *A*_*i*_ and, and individual endowments include asset level, acreage and farming skills.


(10) $$V_{a\,i}\,\left(\omega_{i},f_{i},A_{i},r\right)=u_{a,1}\,\left(\omega_{i},b_{a,i}^{*}\right)+\beta \,u_{a,2}\,\left(f_{i},A_{i},b_{a,i}^{*},r\right)$$

#### 2.2.4 Occupational options

Individuals make decisions based on benefits and costs. If immigration benefits outweigh opportunity costs, then individuals will choose to move. The same is true for the choice of entrepreneurship. We argue that endowments, preference characteristics, and the external environment combine to determine the career choice of individuals. Individuals make occupational options to maximize their effects, and the choice of entrepreneurship and other careers (including work and farming) are mutually opportunity costs. In other words, individuals will only start a business if the benefits of doing so are higher than the benefits of work [refer to Eq. (11)]:


$$V_{e\,i}\,\left(\omega_{i},R_{i},r,\emptyset \right)$$

(11) $$>\text{max}\,\left\{V_{w\,i}\,\left(\omega_{i},w_{i},r\right),V_{a\,i}\,\left(\omega_{i},f_{i},A_{i},r\right)\right\}$$

Individuals are more likely to choose entrepreneurship if it brings more utility and to choose work or farming if the choice brings more benefits. Therefore, we define the individual occupational choice satisfying Eq. (12). We use π in Eq. (13) to measure the difference between the indirect utility of the two choices.


(12) $$E_{i}=\left\{ \begin{array}{c} 1,i\,f\,\pi >0\\ 0,i\,f\,\pi <0 \end{array}\right.$$

$$\pi_{i}=V_{e\,i}\,\left(\omega_{i},R_{i},r,\emptyset \right)$$

(13) $$-\text{max}\,\left\{V_{w\,i}\,\left(\omega_{i},w_{i},r\right),V_{a\,i}\,\left(\omega_{i},f_{i},A_{i},r\right)\right\}$$

Based on the above model, we discuss that factors would impact an individual’s career choice based on comparative static analysis, the individual would choose entrepreneurship when π_*i*_ > 0. Therefore, we can find if π has a positive first-order partial derivative for a given factor, the factor has a positive pro-entrepreneurial effect, otherwise, it will inhibit entrepreneurship.

According to the career choice model, people make their career choices by comparing the utility of different occupations. Moreover, through the career choice model, it is possible to better judge the impact of rural return migrants on entrepreneurship. We argue that the rural return migrants are not only the return of labor itself but also the flow of various factors of production such as knowledge, skills, and capital. The flow of factors brought about by labor mobility optimizes the conditions for residents to start their own businesses, influencing their career choices and, thus, increasing their probability of starting a business. Therefore, we develop the following hypothesis:

Hypothesis 1: Rural return migrants can increase the probability of entrepreneurship among returning labor.

### 2.3 Mechanisms

#### 2.3.1 Land circulation

The difference between rural and urban labor migrants is the land. The opportunity cost of working outside the home is the benefit that farmers can derive from the land. As rural residents own more land, they are less likely to choose to migrate (Hu et al., 2011). In addition, the departure of rural residents, especially young and strong laborers, will lead to the abandonment of the land they would otherwise own. This may result in the outgoing residents renting or even selling the land they own for a higher return, while such laborers will face the dilemma of having less land when they return, directly reducing their probability of farming. Residents who might otherwise choose to work in agriculture give up farming, and this affects their occupational choice as in Eq. (14):


$$\frac{\partial \pi_{i}}{\partial A_{i}}=-\frac{\partial V_{a\,i}\,\left(\omega_{i},f_{i},A_{i},r\right)}{\partial A_{i}}=-\frac{\partial u\,\left(y_{a,i}\right)}{\partial y_{a,i}}\,\frac{\partial y_{a,i}}{\partial A_{i}}$$

(14) $$=-f_{i}\,\frac{\partial u\,\left(y_{a,i}\right)}{\partial y_{a,i}}$$

Where *f*_*i*_ is the individual farming efficiency. We assume that the labor input must lead to the land output, thus, the farming efficiency is constantly positive. According to u(ya,i)′0, so $\frac{\partial \pi_{i}}{\partial A_{i}}<0$ holds constant. Therefore, labor migration may lead to land circulation with a decrease in the amount of arable land for expatriates (Δ*A*_*i*_ < 0) and further promote an increased choice of entrepreneurship among rural return migrants (Δπ_*i*_ > 0). Farmers who work outside have a higher likelihood of transferring their land (Kung, 2002). Decisions to shift labor occur before those to transfer farmland. Thus, the outworking experience significantly increases the probability of farmland transfer, and then increases the entrepreneurial probability. The asset-based income generated from the land circulation, together with the wage income from working outside the home, constitutes physical capital, which in turn contributes to the farmers’ entrepreneurial performance (Yang and Wen, 2020). Therefore, we develop the following hypothesis:

Hypothesis 2.1: Working outside the home will decrease land owned by rural residents.

Hypothesis 2.2: Residents with less land are more likely to start their own businesses.

#### 2.3.2 Human capital

The experience of working outside the workplace allows the workforce to gain skills and experience that help them to build human capital (Xu et al., 2017). Specifically, human capital (*k*_*i*_) can be increased in two ways: (i) an increase in the entrepreneurial talent of managers, leading to an increase in the rate of return per unit of investment in entrepreneurship; (ii) an increase in the efficiency of the workforce and an increase in wages. The combined effects are that the rural return migrants will affect human capital accumulation, which in turn will affect entrepreneurship. We find the partial derivative of the entrepreneurship function (π_*i*_) with respect to human capital (*k*_*i*_), as shown in Eq. (15):


$$\frac{\partial \pi_{i}}{\partial k_{i}}=\frac{\partial V_{e\,i}\,\left(\omega_{i},R_{i},r,\emptyset \right)}{\partial k_{i}}-\frac{\partial V_{w\,i}\,\left(\omega_{i},w_{i},r\right)}{\partial k_{i}}$$

$$=p\,\beta \,\frac{\partial u_{e,2}\,\left(R_{i},r\right)}{\partial k_{i}}-\beta \,\frac{\partial u_{w,2}\,\left(w_{i},r\right)}{\partial k_{i}}$$

(15) $$=p\,\beta \,\frac{\partial u\,\left(y_{s,i}\right)}{\partial y_{s,i}}\,\frac{\partial y_{s,i}}{\partial R_{i}}\,\frac{\partial R_{i}}{\partial k_{i}}-\beta \,\frac{\partial u\,\left(y_{w,i}\right)}{\partial y_{w,i}}\,\frac{\partial y_{i}}{\partial w_{i}}\,\frac{\partial w_{i}}{\partial k_{i}}$$

We can find that the magnitude of the effect of human capital accumulation on residents’ entrepreneurial motivation depends on the growth of entrepreneurial income versus wage income as a result of human capital accumulation. Outgoing labor types, the different industries they work in, or the position types they hold will impact individual human capital (Shi and Zhou, 2007; Lee, 2018; Tajpour and Hosseini, 2019). Generally, production activities could increase labor efficiency and management activities increase entrepreneurial talents. The exact increase magnitude is determined by the production and management share of the job. We argue that if rural laborers are engaged in management and administration before they return home, the human capital accumulation will lead to an increase in their entrepreneurial talent and no significant change in individual labor efficiency ($\frac{\partial R_{i}}{\partial k_{i}}>0,\frac{\partial w_{i}}{\partial k_{i}}=0$). If rural laborers are engaged in productive work, their entrepreneurial talent will not be accumulated, but labor efficiency will be improved and their wages will rise after they return home ($\frac{\partial R_{i}}{\partial k_{i}}=0,\frac{\partial w_{i}}{\partial k_{i}}>0$). Existing studies state that farmers with outworking experience have broader social networks, based on which they can harvest more capital, more customers, and more convenient business permits for entrepreneurship (Xu et al., 2017). The outworking experience significantly improves the probability of starting a business by increasing the human capital accumulation and financing sources for entrepreneurship (Zhou et al., 2017). Therefore, we develop the following hypothesis:

Hypothesis 3.1: Rural return migrants can increase human capital accumulation.

Hypothesis 3.2: The accumulation of human capital will lead to an increase in productivity and managerial capacity, which together influence the entrepreneurial choices of residents.

#### 2.3.3 Physical capital

The lack of capital has always been a major disincentive to entrepreneurship (Batjargal, 2007; Hrytsaienko et al., 2019), and the accumulation of physical capital brought about by the return of labor may affect the motivation of the workforce to start their own businesses. We show the effect using the partial derivative of the entrepreneurship function (π_*i*_) with respect to physical capital (ω_*i*_), as shown in Eq. (16). The first term in Eq. (16) is the marginal utility from the first period of increased consumption when choosing to start a business, and the second term is the marginal utility from the first period of increased consumption when choosing to work:


$$\frac{\partial \pi_{i}}{\partial \omega_{i}}=\frac{\partial V_{e\,i}\,\left(\omega_{i},R_{i},r,\emptyset \right)}{\partial \omega_{i}}-\frac{\partial V_{w\,i}\,\left(\omega_{i},w_{i},r\right)}{\partial \omega_{i}}$$

$$=\frac{\partial u\,\left(x_{e,i}\right)}{\partial x_{e,i}}\,\frac{\partial x_{e,i}}{\partial \omega_{i}}-\frac{\partial u\,\left(x_{w,i}\right)}{\partial x_{w,i}}\,\frac{\partial x_{w,i}}{\partial \omega_{i}}$$

(16) $$=\frac{\partial u\,\left(x_{e,i}\right)}{\partial x_{e,i}}-\frac{\partial u\,\left(x_{w,i}\right)}{\partial x_{w,i}}$$

The magnitude of both will influence the effect of physical capital on entrepreneurial motivation, i.e., depending on individual endowments and preferences (Arafat et al., 2020). Increases in physical capital do not directly promote or inhibit the probability of individual entrepreneurship but rather vary according to individual differences. Therefore, we develop the following hypothesis:

Hypothesis 3.1: Rural return migrants can lead to the accumulation of physical capital.

Hypothesis 3.2: An increase in physical capital will increase the utility of all career choices, and whether or not it has a facilitating effect on entrepreneurship depends on the size of the increase in utility for different career types.

### 2.4 Entrepreneurial effects of rural return migrants

From the above analysis, we argue that the rural return migrants lead to an increase in the probability of entrepreneurship, expressed as the derivative of the occupational choice function (π) with respect to the return of resident labor (*M*). We denote the entrepreneurial effect of rural return migrants as *T*(*M*), as shown in Eq. (17):


$$T\,\left(M\right)=\frac{\partial \pi }{\partial M}=\frac{\partial \pi }{\partial k}\,\frac{\partial k}{\partial M}+\frac{\partial \pi }{\partial \omega }\,\frac{\partial \omega }{\partial M}+\frac{\partial \pi }{\partial A}\,\frac{\partial A}{\partial M}$$

$$=\left[p\,\beta \,\frac{\partial u\,\left(y_{s,i}\right)}{\partial y_{s,i}}\,\frac{\partial y_{s,i}}{\partial R_{i}}\,\frac{\partial R_{i}}{\partial k_{i}}-\beta \,\frac{\partial u\,\left(y_{w,i}\right)}{\partial y_{w,i}}\,\frac{\partial y_{i}}{\partial w_{i}}\,\frac{\partial w_{i}}{\partial k_{i}}\right]\,\frac{\partial k}{\partial M}$$

(17) $$+\left[\frac{\partial u\,\left(x_{e,i}\right)}{\partial x_{e,i}}-\frac{\partial u\,\left(x_{w,i}\right)}{\partial x_{w,i}}\right]\,\frac{\partial \omega }{\partial M}-\left[f_{i}\,\frac{\partial u\,\left(y_{a,i}\right)}{\partial y_{a,i}}\right]\,\frac{\partial A}{\partial M}$$

The entrepreneurial effects of rural return migrants are divided into three categories. The first term, outside the brackets, is the human capital growth brought about by the rural return migrants while the inner bracket is the impact of the human capital growth per unit on entrepreneurship, and the product of the two is the impact of the rural return migrants on entrepreneurship through the human capital path. Similarly, the second and third terms represent the impact of the rural return migrants on entrepreneurship through physical capital accumulation and land circulation, respectively. This implies that the entrepreneurial effect of rural return migrants is the result of the combined effect of human capital, physical capital, and land circulation.

## 3. Methodology

### 3.1 Sample and data

First, we use micro-individual data. Current literature mainly uses macro (Zhang and Cen, 2014; Yingen and Guangli, 2020), micro (Oostendorp, 2017), and big data (Obschonka and Audretsch, 2020). The main reason for abandoning the use of macro data is its difficulty to access. At present, direct macro statistics on population movements are still scarce. Existing studies using macro data are mainly based on “historical census data” (Zhang and Cen, 2014) or use “current resident population –(1 + r) × previous resident population” (Yang and Wen, 2020). This is slightly simplistic and crude. There are also some newer studies that use big data methods to count population movements (Obschonka and Audretsch, 2020), but this measurement is even less applicable to the topic. The reasons for this are: first, this measurement is more ambiguous. Big data reflect an offset of population inflows and outflows, but we focus labor force return, which required defining accurately population inflows and outflows. This measurement approach is very crude. The population flows obtained in this way are only meaningful in terms of current flows, whereas labor returns are a long-term effect and are not suitable for measurement using flow data. Therefore, it is more appropriate to use micro data for this analysis.

Then, we use cross-sectional data. Panel data are the dominant research basis in most existing research, but the use of cross-sectional data in our research is based on two considerations: (i) the way labor returns are asked in the questionnaire is inconsistent across years (Xu et al., 2017), which does not accurately meet the single definition in our research; (ii) changes in the indicator of “whether or not one is a returnee” over time are not obvious in the questionnaires of different years. Only samples that change from non-returning to returning individuals between surveys are valid, and the use of panel fixed effects results in a large number of missing samples. Therefore, following Shi and Yang (2012) and Zhou et al. (2017), we use cross-sectional data for the analysis.

The data are mainly from the CLDS and CHFS, which are used widely (Zhou et al., 2017; Yang and Wen, 2020). The CLDS survey covers all labor force members aged 15–64 in the surveyed households, focusing on the labor force characteristics of respondents, which facilitates further exploration of rural return migrants. We used “whether the rural sample has experience of working outside” as the criterion to determine whether the sample belongs to the labor force return, and “whether the sample has work experience” to determine whether the sample belongs to the labor force, and finally obtained 5,591 samples. The CHFS focuses on the financial attributes of respondents, providing a wealth of data on entrepreneurship, which provides good conditions for analysis at the entrepreneurial level. We use only the data from CHFS to test for the mediating effect of physical capital and obtain 29,339 samples. It was not possible to match the two databases because of the different respondent groups and different coding methods. However, in the empirical part, using two different databases for the study also allows cross-validation, making the results more robust and more credible.

### 3.2 Variables measurement

#### 3.2.1 Dependent variables

Entrepreneurship. We use the question “Was the last job an entrepreneur?” to measure whether the sample is entrepreneurial, denoted by *E*. *E* is 1 for entrepreneurs and 0 otherwise.

#### 3.2.2 Independent variables

Rural return migrants. We consider that rural return migrants need to meet three conditions: (i) having migrant experience; (ii) Being rural samples; and (iii) being of working age. We define samples as rural return migrants that satisfy the above conditions, denoted by *mig*. *mig* is 1, 0 otherwise.

#### 3.2.3 Control variables

Following existing literature, we set control variables at the individual, household, and village levels that may affect entrepreneurial behavior in Supplementary Appendix 2.

### 3.3 Empirical models

To examine the direct entrepreneurial effects of rural return migrants, we build the Probit model in Eq. (18) based on the theoretical analysis and research hypothesis in section 2 “Theoretical models and hypotheses,” considering that the explanatory variable “resident entrepreneurship” is a 0–1 variable:


(18) $$P\,r\,\left(E_{i}\right)=\alpha +\delta \,m\,i\,g_{i}+\Gamma \,X_{i}+\varepsilon_{i}$$

Where *E*_*i*_ is entrepreneurship. *mig*_*i*_ is the dummy for rural return migrants, and it is the core explanatory variable whose coefficient is denoted by δ. *X*_*i*_ stands for a series of control variables (Refer to Supplementary Appendix 2). ε_*i*_ is residual. If δ is significantly positive, it indicates that the return of rural labor has a positive effect on rural entrepreneurship.

## 4. Results

### 4.1 Descriptive statistics

Table 1 presents descriptive statistics and shows that 1.04% of the sample start their own business and 23.86% are rural return migrants. The basic profile of the variables is consistent with the distribution: the sample surveyed was between 15 and 91 years old, with an average age of 50 years. The female sample is slightly larger, but the gender ratio is generally more balanced, in line with the aging and feminization of rural areas. The rest of the variables are distributed more normally. Therefore, this paper considers the selection of the sample to be somewhat representative.

**TABLE 1:** Statistics description.

Variable	*N*	Mean	Std. dev.	Min	Max
*E*	5,960	0.0104	0.1015	0	1
*mig*	5,960	0.2386	0.4263	0	1
Age	5,951	50.5646	12.6684	15	87
Gender	5,960	1.4651	0.4988	1	2
Marital status	5,960	2.1047	0.7415	1	6
Political affiliation	5,952	2.9059	0.4231	1	3
Education	5,955	2.7081	1.5239	1	10
Health	5,958	2.4651	1.0070	1	5
Household size	5,960	1.6951	0.3600	0.6931	2.9444
Household savings	5,953	1.9189	0.2731	1	2
Household income	5,647	9.9103	2.0391	0.0000	14.2210
Village size	5,960	6.3395	0.7765	4.0431	9.0807
Village location	5,960	3.0243	0.8444	0	5.70711
Village level	5,960	1.8534	0.3538	1	2

There are slight differences in the sample sizes of the variables due to missing variables. We logarithmise the two metrics are not applicable to our research amount. Core explanatory and explained variables are 1 for yes and 0 for no. Control variables are 1 for yes and 2 for no.

### 4.2 Empirical results analysis

Table 2 reports the results of tests of the entrepreneurial effect of the rural return migrants. Without considering control variables, the coefficients of *mig*_*i*_ in columns (1) is 0.3688 and significant at the 1% level. This means rural return migrants are 36.88% more likely to start a business than rural residents on average. The coefficients of *mig*_*i*_ in columns (2)–(4) are 0.2690, 0.3238, and 0.3491, respectively, controlling for individual, household, and village characteristics of the sample and are significant at least at the 5% level. We believe that the rural return migrants have a higher incentive to start a business, and the results are somewhat robust as they still hold control over other variables.

**TABLE 2:** Regression results.

Variables	(1)*E*	(2)*E*	(3)*E*	(4)*E*
*mig*	0.3688*** (0.101)	0.2690** (0.112)	0.3238*** (0.125)	0.3491*** (0.123)
Age		−0.0087** (0.004)	−0.0071 (0.005)	−0.0069 (0.005)
Gender		−0.3558*** (0.110)	−0.3941*** (0.128)	−0.3982*** (0.129)
Marriage		−0.0854* (0.045)	−0.0822 (0.056)	−0.0868 (0.056)
Political affiliation		0.0492 (0.124)	0.0559 (0.123)	0.0572 (0.120)
Education		0.0629** (0.028)	−0.0286 (0.033)	−0.0335 (0.034)
Health		−0.1187** (0.060)	−0.0406 (0.073)	−0.0334 (0.074)
Household size			−0.2330 (0.160)	−0.2263 (0.163)
Household savings			−0.3802** (0.149)	−0.4159*** (0.154)
Household income			0.5132*** (0.073)	0.4996*** (0.073)
Village size				0.0445 (0.061)
Village location				−0.1105* (0.060)
Village level				−0.0057 (0.161)
Con	−2.4169*** (0.060)	−1.3900*** (0.524)	−5.8274*** (0.910)	−5.5870*** (1.025)
*N*	5,960	5,936	5,621	5,591
*R* ^2^	0.0181	0.0759	0.192	0.197

Supplementary Appendix 2 provides definitions of control variables. Robust standard errors in brackets, ***, **, and * denote significance at the 1, 5, and 10% statistical levels, respectively.

### 4.3 Robustness checks

#### 4.3.1 Alternative measurement

First, we use the migration experience to re-measure rural return migrants, dividing the cross-county sample into those with cross-township and those with non-township migration experiences. We also use the self-employed to re-measure entrepreneurship. We believe that entrepreneurship is defined as a person who makes an initial investment in the business and can bear the returns and risks. Therefore, we relax the original entrepreneurship condition by defining both “employer” and “Self-employed” as entrepreneurs (the original indicator only defined employers as entrepreneurs, expressed as “Business”) and define other employment statuses as non-entrepreneurs. Robustness test results in Table 3 show that the results are still significant at least at the 5% level, and the coefficients of *mig* and migration experience are around 0.3 migration experience, which consists of previous empirical research results.

**TABLE 3:** Alternative measurement.

Variables	Business		Self-employment	
	(1)*E*	(2)*E*	(3)*E*	(4)*E*
*mig*	0.3491*** (0.123)		0.2603*** (0.060)	
Migration experience		0.3013** (0.119)		0.2181*** (0.058)
Controls	Yes	Yes	Yes	Yes
Con	−5.5870*** (1.025)	−5.5600*** (1.022)	−2.0854*** (0.514)	−2.0821*** (0.514)
*N*	5,591	5,591	5,591	5,591
*R* ^2^	0.197	0.195	0.0749	0.0735

Robust standard errors in brackets, ***, **, and * denote significant at the 1, 5, and 10% statistical levels, respectively.

#### 4.3.2 Alternative models

Considering that there may be cases where the sample does not meet the assumptions of the Probit regression, we estimate the coefficients using alternative estimation methods such as Logit, OLS, and GMM. From the robustness test results in Table 4, we can see that changing the models does not affect the previous empirical research results.

**TABLE 4:** Alternative models.

Variables	Probit	Logit	OLS	GMM
	(1)*E*	(2)*E*	(3)*E*	(4)*E*
*mig*	0.3491*** (0.123)	0.7783*** (0.301)	0.0077** (0.003)	0.0077* (0.004)
Controls	Yes	Yes	Yes	Yes
Con	−5.5870*** (1.025)	−12.6021*** (2.387)	0.0306 (0.023)	0.0306 (0.023)
*N*	5,591	5,591	5,591	5,591
*R* ^2^	0.197	0.196	0.014	\

Robust standard errors in brackets, ***, **, and * denote significant at the 1, 5, and 10% statistical levels, respectively.

#### 4.3.3 Sample changes

We use different criteria to redefine the workforce. First, we use the working experience as a criterion and consider that the sample only needs to meet the requirement of having work experience without age limits. We then redefine the workforce by working population aged 15–64 and with working experience. Retirement age is the third criterion, and the sample is not below the legal retirement age in the current year, but also with working experience.^1^ The robustness results are shown in Table 5 and are consistent with previous empirical research results.

**TABLE 5:** Sample changes.

Variables	Working experience	Working population	Retirement age
	(1)*E*	(2)*E*	(3)*E*
*mig*	0.3491*** (0.123)	0.3661*** (0.125)	0.3266** (0.131)
Controls	Yes	Yes	Yes
Con	−5.5870*** (1.025)	−5.7787*** (1.091)	−5.7527*** (1.157)
*N*	5,591	4,877	3,495
*R* ^2^	0.197	0.191	0.165

Robust standard errors in brackets, ***, **, and * denote significance at the 1, 5, and 10% statistical levels, respectively.

### 4.4 Endogeneity

#### 4.4.1 Instrumental variable regression

To further alleviate endogeneity caused by reverse causality and omitted variables, we use village returners’ proportion as an instrumental variable (Wahba and Zenou, 2012) in a 2SLS regression to address endogeneity. Both outworking and returning home have network effects, especially in rural areas. One person’s going out to work (returning home) may lead to others going out (returning home). Therefore, we believe that the proportion of village returners is correlated with entrepreneurship. Furthermore, the return rate of villages as a whole does not affect the probability of individual residents starting a business. The results of the two-stage instrumental variable regression are shown in Table 6. Panels A and B show the second and first-stage regressions, respectively. Column (1) shows the results of the instrumental variables regression in the base regression, and columns (2)–(4) show the results of robustness tests using different measures of entrepreneurship and rural return migrants, respectively. Panel B shows Residents of regions with higher rates of return have a stronger propensity to return, satisfying the correlation hypothesis of the instrumental variable. Panel A shows the coefficients of *mig* and migration experience both remain significant and positive. The results indicate that our findings do not appear to be driven by endogeneity.

**TABLE 6:** IV- 2SLS.

Variables	Business	Self-employment	Business	Self-employment
	(1)*E*	(2)*E*	(3)*E*	(4)*E*
**Panel A: second stage**				
*mig*	0.6461* (0.389)	0.8414*** (0.169)		
Migration experience			0.6517* (0.374)	0.8264*** (0.163)
Controls	Yes	Yes	Yes	Yes
Con	−5.6829*** (1.131)	−2.2186*** (0.474)	−5.6705*** (1.123)	−2.2268*** (0.471)
**Panel B: first stage**				
Returners proportion	0.9881*** (0.036)	0.9881*** (0.036)	1.0091*** (0.038)	1.0091*** (0.038)
Controls	Yes	Yes	Yes	Yes
Con	0.4582*** (0.086)	0.4582*** (0.086)	0.4874*** (0.091)	0.4874*** (0.091)
*N*	5,591	5,591	5,591	5,591

Robust standard errors in brackets, ***, **, and * denote significance at the 1, 5, and 10% statistical levels, respectively.

#### 4.4.2 Propensity score matching (PSM)

We used all the control variables in the previous section as covariates and matched them using kernel matching, nearest neighbor matching, and caliper matching, respectively, and regressed the matched samples.^2^ The results in Table 7 show that there is a significant difference in the probability of starting a business between the treatment and control groups, regardless of the matching method used. Moreover, the effect of rural return migrants on resident entrepreneurship remains positively significant when regressed using the matched samples. With the exclusion of sample self-selection bias, the results remain largely unchanged, and rural return migrants are a significant boost to resident entrepreneurship.

**TABLE 7:** Propensity score matching results.

Variables	Business			Self-employment		
	Kernel	Nearest neighbor (*n = 5)*	Caliper (*cal* = 0.05)	Kernel	Nearest neighbor (*n = 5)*	Caliper (*cal* = 0.05)
	(1)*E*	(2)*E*	(3)*E*	(4)*E*	(5)*E*	(6)*E*
*ATT*	0.0076	0.0088	0.0075	0.0366	0.0416	0.0368
*t*	1.75	1.90	1.75	3.48	3.68	3.50
*mig*	0.2881** (0.119)	0.3622*** (0.131)	0.2891** (0.119)	0.2613*** (0.062)	0.3622*** (0.131)	0.2891** (0.119)
Controls	Yes	Yes	Yes	Yes	Yes	Yes
Con	−6.4562*** (1.225)	−7.0850*** (1.332)	−6.4616*** (1.225)	−2.7067*** (0.628)	−7.0850*** (1.332)	−6.4616*** (1.225)
*N*	5,589	3,750	5,589	5,589	3,750	5,589
*R* ^2^	0.152	0.180	0.152	0.0670	0.180	0.152

Robust standard errors in brackets, ***, **, and * denote significance at the 1, 5, and 10% statistical levels, respectively. The explanatory variables in columns (1)–(3) are business (consider employing others as entrepreneurship only); columns (4)–(6) are self-employment (consider both employment and self-employment as entrepreneurship).

## 5. Mechanisms

### 5.1 Land circulation

We use the *land* to denote land transfers, measured by the area of land owned by individuals and logged. Following Baron and Kenny (1986), we build on the previous findings by conducting a further test, the results are shown in Table 8. In column (1), the coefficient of *mig* is −0.0936 and significant at a 1% level, indicating that rural return migrants with experience of working outside the home tend to have less land per capita than other residents and that there is land circulation among rural return migrants. In column (2), the coefficient of *mig* is significantly positive, and the coefficient of *land* is significantly negative, indicating that the rural return migrants can promote entrepreneurship through land circulation.

**TABLE 8:** Mechanisms.

Variables	Land circulation		Human capital		Physical capital		
	(1)*land*	(2)*E*	(3)*abli*	(4)*E*	(5)*E*	(6)*save*	(7)*E*
*mig*	−0.0936*** (0.027)	0.3134** (0.126)	0.0348*** (0.009)	0.3395** (0.132)	0.1580*** (0.043)	0.1763*** (0.061)	0.1646*** (0.046)
*land*		−0.1833** (0.092)					
*abli*				0.0763* (0.040)			
*save*							0.0248*** (0.006)
Controls	Yes	Yes	Yes	Yes	Yes	Yes	Yes
Con	3.1818*** (0.201)	−5.3772*** (1.154)	4.2096*** (0.378)	−5.6972*** (1.123)	−1.5664*** (0.230)	7.8901*** (0.298)	−1.6806*** (0.259)
*N*	5,539	5,539	4,644	4,644	40,697	29,339	29,339
*R* ^2^	0.109	0.229	0.063	0.192	0.090	0.081	0.086

Robust standard errors in brackets, ***, **, and * denote significance at the 1, 5, and 10% statistical levels, respectively.

### 5.2 Human capital

Human capital is a factor of entrepreneurship and measures human capital in terms of individual competencies (Xu et al., 2017). An individual’s ability is matched by the difficulty of competency in a job. As an individual’s ability increases, the individual can obtain a job that matches his or her ability through promotion, job-hopping, and changing job content. We thus equate job difficulty with individual ability, denoted as *abli* and include it as a mediating variable in the regression model.

Table 8 reports the results of the mechanism test for human capital. The coefficient of *mig* in column (3) is 0.0348 and significant at 1% level, indicating that the experience of working away from home has led to a higher level of personal competence in the returning workforce, and it is reflected in the increased difficulty in performing the job. Column (4) incorporates both the core explanatory variables and the mediating variables into the regression model. The coefficient of *mig* is significantly positive and the coefficient of *abli* in column (4) is significantly negative. The results show that the effects of rural return migrants on entrepreneurship remain significant when also controlling for individual capabilities, suggesting that rural return migrants still have a facilitating effect on entrepreneurship and that human capital plays a part in mediating the effect.

### 5.3 Physical capital

We use *save* to measure the physical capital formed by rural return migrants working outside the home. In China, the vast majority of entrepreneurship and work is a household activity, so the use of household savings provides some indication of an individual’s physical capital, and the results obtained using household data are credible. We, thus, use the balance of savings in a current account to measure *save*. To mitigate problems such as heteroskedasticity, we add 1 to it and take the logarithm. Columns (5)–(7) in Table 8 show the results of the test for the mediating effect of physical capital. The results in column (5) show that the rural return migrants have a higher entrepreneurial motivation with a coefficient of 0.1580. The coefficient of *mig* is significantly positive in columns (6) and suggests rural return migrants tend to have higher physical capital. Specifically, it is 0.1763% higher and significant at the 1% level. Column (7) explores the effect of rural return migrants on entrepreneurial intentions, controlling for physical capital, and finds that the results remain significant. This suggests that while physical capital plays a mediating role, again it is only partially mediated.

## 6. Conclusion and implications

Entrepreneurship is generally recognized as a key component in the development process and especially a scarce resource in economically disadvantaged rural areas. The return of rural labor outside the home is related to the promotion of urbanization and the implementation of the rural revitalization strategy and is of great importance to regional economic development. We first theoretically reveal the mechanism of occupational choice and the entrepreneurial propensity of rural return migrants under different occupational returns by constructing an occupational choice model of rural return migrants, and then empirically test the relationship between rural return migrants and entrepreneurial effect using CLDS (2018) and CHFS (2019). The results show that rural return migrants have a positive effect on entrepreneurship, and this effect still holds when controlling for the remaining variable and this finding holds after accounting for endogeneity. We further find that land circulation, human capital, and physical capital are stimulating factors in promoting rural entrepreneurial activities of rural return migrants, and there is a threshold effect on physical capital.

Based on the results, we put forward the following targeted policy recommendations for the rural labor return in China: first, following the trend of labor force return, the base of the entrepreneurial group should be enlarged. The government should seize the opportunity of rural revitalization, build a platform for employment and entrepreneurship based on industrial development, and expand the development space with entrepreneurship support policies as a guarantee. The aging of rural areas is becoming increasingly serious and fertility growth is not promising. The government should take measures to increase the birth rate in rural areas and strengthen the security of retirement, health care, and education in rural areas to provide the basis for a larger rural entrepreneurial group.

Second, it is important to improve the land circulation model and increase the willingness of rural residents to start their businesses. Establish a clear system of property rights and adhere to, consolidate, and improve relevant land policies. Meanwhile, the market-based mechanism of land circulation should be used rationally, and the owners of rural land should be actively guided to use market-based means to obtain the proceeds of land circulation. It is important to strike a balance between efficiency and fairness in the process of land transfer and to prevent the polarization of the income of rural residents. The direction of land circulation is to obtain a continuous increase in marginal returns through increased productivity and industrial development on the premise of a moderate scale.

Third, human capital should be strengthened to provide intellectual support for rural residents to start their businesses. At present, the education, skill, and business management levels of rural labor are low as a whole. The lack of human capital will limit the willingness of rural residents to start their businesses and the scale of entrepreneurship. For the rural return migrants, improving human capital is not a short-term quick fix, and the government should pay attention to it at all stages. It is necessary to increase financial investment in basic education in rural areas, strengthen the policy inclination for rural students in vocational and higher education, and encourage social forces to participate in rural education in various forms.

Finally, the accumulation of physical capital should be valued to provide sufficient funds for rural residents to start their businesses. The government should take measures to narrow the income gap within rural areas and broaden the sources of income for rural residents through market-based means. Meanwhile, the government should encourage rural residents to expand their consumption and improve their consumption structure. The government should tap and release the huge consumption potential in rural areas so that consumption can drive production, and production can drive investment and entrepreneurship.

Policymakers should consider the psychological effects of changes in the economic environment of migrant workers who go out to work and the economic consequences of their return to rural. Entrepreneurship should be paid attention to as an important tool to drive rural economic development. Neglecting rural migrants’ entrepreneurship is not conducive to sustainable rural development. We are committed to developing a complete and insightful understanding of rural return migrants and their entrepreneurial effects. However, it must be acknowledged that the theoretical and empirical analysis in this paper has been done in the context of a low volume of relevant literature and insufficient experience, due to the relatively small amount of existing research and experience. Data on rural return labor have limitations at both the macro and micro levels. Due to a lack of statistical data, we are unable to provide an accurate count of the number of people in this group. We, therefore, expect to make greater use of mathematical language in future research to complete the derivation of the entrepreneurial effects of rural return labor and to provide scientific evidence to support this topic. Entrepreneurship is becoming increasingly important in economic development, and there are differences in rural development across China and around the world. We believe that this theme will be a hot topic for academic research, with a vertical analysis of the time characteristics and a horizontal analysis of the regional differences, as well as an analysis of how to support and protect the entrepreneurial behavior of rural return migrants on the basis of the economic and social risks arising from large-scale population movements.

## Data availability statement

The data analyzed in this study is subject to the following licenses/restrictions: The data will be used in subsequent studies. Requests to access these datasets should be directed to corresponding author.

## Author contributions

AB and GP contributed to the conception, design, and formal analysis of the study. AB performed the methodology, software, data curation, and writing – original draft preparation. GP performed the validation, investigation, and writing – review and editing. GZ performed the writing – review and editing and supervision. All authors contributed to manuscript revision, read, and approved the submitted version.

## Footnotes

1 The regulations in 2018 provide for retirement at age 60 for men and 50 for women.

2 The results of our tests using nearest neighbor sizes (n = 3, 4, 5, 6) and caliper values (cal = 0.03, 0.04, 0.05, 0.06) remain robust and are not listed here due to space constraints and are available upon request.
