# Supplementary material for: Entrepreneurial effect of rural return migrants: Evidence from China
Source: Front Psychol. 2022 Dec 22;13:1078199. doi: 10.3389/fpsyg.2022.1078199 (PMC9815529; doi:10.3389/fpsyg.2022.1078199)
Supplement: Supplementary file 1 [file Data_Sheet_1.docx]

## Appendix 1.

**Variable Description**

| Variables | Symbols | Definition |
| --- | --- | --- |
| Personal endowment: Exogenous | | |
| Physical capital | $\omega_{i}$ | Sum of all assets owned in the period |
| Human capital | $k_{i}$ | Personal talent, including business talent and work talent |
| Loan limits | $\bar{b}_{i}$ | Maximum amount of funding an individual can borrow |
| Discount rate | $\beta$ | $Preference for future funding, \beta\in(0.1)$ |
| Entrepreneurial ability | $R_{i}$ | Entrepreneurial talent, influenced by human capital |
| Wages | $w_{i}$ | Remuneration through labour, influenced by human capital |
| External environment: Exogenous | | |
| Loan interest rate | $r$ | Costs paid for the loan, repaid in the second installment |
| Entrepreneurial residuals | $\emptyset$ | Capital residuals over after a failed venture |
| Entrepreneurial probability | $p$ | Probability of success in business under the current condition |
| Personal Choice: Endogenous | | |
| Loan amount | $b_{i}$ | For inter-period allocation of funds, no higher than the loan limit |
| Investment amount | $m_{i}$ | Entrepreneurial capital, not exceeding the total funds available |
| Current consumption | $x_{i}$ | Consumption in the first period |
| Future consumption | $y_{i}$ | Consumption in the second period |
| Total utility | $U_{i}$ | Determined by two-period utility and discount rate |
| Indirect utility | $V_{i}$ | Utility other than consumption |
| Category variable | | |
| Individual | $i$ | Subscript denotes the i-th individual |
| Occupation | $e,w$ | Subscript, $e$ denotes entrepreneurship and $w$ denotes wage |
| Entrepreneurship | $s,f$ | Subscript, $s$ denotes a successful entrepreneurship and otherwise $f$ |

*Note:* Less important variables are described in the paper.

## Appendix 2.

**Control variable description**

| Variables | Definition | |
| --- | --- | --- |
| Individual characteristics | | |
| Gender | | Respondents' gender; 1=male, 2=female |
| Age | | Respondents' age in the year |
| Political affiliation | | Respondents' political affiliation, party 1=member, 2=non-party member |
| Health | | Respondents' health status self-assessment, healthy, good, fair |
| Education | | Respondent's highest level of education |
| Marital status | | Single, divorced, widowed, etc. |
| Family characteristics | | |
| Household income | | Total household income of surveyed households for the year |
| Household savings | | Whether surveyed households have money to lend to others |
| Household size | | Number of people in surveyed household |
| Village characteristics | | |
| Village size | | Total number of households in the interviewed village |
| Village location | | Distance between the surveyed village and the nearest county/district government |
| Village level | | Whether the village is the seat of the township government |

*Note:* in core explanatory and explained variables, 1 means yes, and 0 means no. In control variables, 1 means yes, and 2 means no.
